# Therapeutic strategies for cell-based neovascularization in critical limb ischemia

**DOI:** 10.1186/s12967-017-1153-4

**Published:** 2017-02-24

**Authors:** Makoto Samura, Tohru Hosoyama, Yuriko Takeuchi, Koji Ueno, Noriyasu Morikage, Kimikazu Hamano

**Affiliations:** 1 Division of Vascular Surgery, Department of Surgery and Clinical Science, Yamaguchi University Graduate School of Medicine, 1-1-1 Minami-kogushi, Ube, Yamaguchi, 755-8505 Japan; 2 Center for Regenerative Medicine, Yamaguchi University Graduate School of Medicine, 1-1-1 Minami-kogushi, Ube, Yamaguchi, 755-8505 Japan; 3 Department of Surgery and Clinical Science, Yamaguchi University Graduate School of Medicine, 1-1-1 Minami-kogushi, Ube, Yamaguchi, 755-8505 Japan; 4 Center for Regenerative Medicine, Department of Surgery and Clinical Science, Yamaguchi University Graduate School of Medicine, 1-1-1 Minami-kogushi, Ube, Yamaguchi, 755-8505 Japan

**Keywords:** Critical limb ischemia, Cell-based therapeutic angiogenesis, Peripheral blood mononuclear cells, Clinical trials, Hypoxic preconditioning, Combination therapy

## Abstract

Critical limb ischemia (CLI) causes severe ischemic rest pain, ulcer, and gangrene in the lower limbs. In spite of angioplasty and surgery, CLI patients without suitable artery inflow or enough vascular bed in the lesions are often forced to undergo amputation of a major limb. Cell-based therapeutic angiogenesis has the potential to treat ischemic lesions by promoting the formation of collateral vessel networks and the vascular bed. Peripheral blood mononuclear cells and bone marrow-derived mononuclear cells are the most frequently employed cell types in CLI clinical trials. However, the clinical outcomes of cell-based therapeutic angiogenesis using these cells have not provided the promised benefits for CLI patients, reinforcing the need for novel cell-based therapeutic angiogenesis strategies to cure untreatable CLI patients. Recent studies have demonstrated the possible enhancement of therapeutic efficacy in ischemic diseases by preconditioned graft cells. Moreover, judging from past clinical trials, the identification of adequate transplant timing and responders to cell-based therapy is important for improving therapeutic outcomes in CLI patients in clinical settings. Thus, to establish cell-based therapeutic angiogenesis as one of the most promising therapeutic strategies for CLI patients, its advantages and limitations should be taken into account.

## Background

Peripheral artery disease (PAD), also called peripheral vascular disease, is characterized by the narrowing of blood vessels, which leads to impaired blood supply to the organs. PAD is caused mostly by atherosclerosis obliterans (ASO) and thromboangiitis obliterans (TAO). Owing to changes in lifestyle, the number of TAO patients is decreasing, while that of ASO patients is increasing. Consequently, as PAD is thought to develop mostly from ASO, the worldwide prevalence of PAD is expected to increase [1].

Critical limb ischemia (CLI) is clinically defined as the chronic and severe stagnation of limb perfusion, its ultimate outcomes being tissue ulceration and gangrene. CLI is commonly caused by PAD and is the disease of arteries of all range size. It can cause diabetic microangiopathy and vasculitis, and is associated with a high risk of cerebro-cardiovascular events, including myocardial infarction and stroke. Accordingly, it presents poor prognosis and high mortality: 20% within 6 months and 50% within 5 years of the diagnosis [2–4]. Surgical bypass and angioplasty for limb revascularization are the gold standards for CLI. However, about 20–30% of patients with CLI are ineligible for these therapies because of severe calcification of the arteries, lack of suitable target arteries and vein graft, and extensive comorbidities [5, 6]. Unfortunately, major limb amputation is required within 1 year for as many as 40% of untreatable CLI patients [3, 7]. Consequently, the development of alternative therapeutic strategies for these high-risk patients is strongly desired.

Therapeutic angiogenesis, which can be induced by delivery of protein(s), gene(s), or cell(s) to ischemic tissues, offers the possibility of blood flow recovery in ischemic limbs, thus sparing CLI patients from major limb amputation [8, 9]. For example, gene delivery of vascular endothelial growth factor (VEGF) resulted in a significant improvement in hemodynamics and skin ulcer in CLI patients, even though there was no significant reduction in the amputation rate after 100 days of treatment [10]. This small randomized trial introduced the possibility of gene delivery-mediated therapeutic angiogenesis for CLI. However, virus-mediated gene therapy often supplies a transient excess of pro-angiogenic factors to ischemic and non-ischemic tissues. This increases the risk of side effects such as malignant alteration of tumors [11]. Although rapid and remarkable advances have been made in gene therapy, suitable gene delivery methods should be developed to reduce excessive pro-angiogenic factors in clinical settings. In contrast, cell delivery strategies, namely cell-based therapies, enable the stable supply of growth factors/cytokines for angiogenesis of ischemic tissues [12]. Particularly, the discovery of endothelial progenitor cells (EPCs) in bone marrow and their strong angiogenic potential encouraged many groups, including ours, to attempt cell-based therapeutic angiogenesis in CLI patients [13–19]. We performed the first human trial of transplantation of bone marrow-derived cells, also known as bone marrow mononuclear cells (BMMNCs), into CLI patients. Even though it involved only a small number of subjects, the procedure demonstrated the feasibility of cell-based therapeutic angiogenesis in CLI patients [20]. Thereafter, many institutions have performed clinical trials using bone marrow-derived cells in CLI patients. In recent years, peripheral blood mononuclear cells (PBMNCs) have also been used for cell-based therapeutic angiogenesis in CLI patients. PBMNCs can be more easily and safely isolated from patients than BMMNCs, while displaying similar therapeutic efficacy [21]. To date, BMMNCs and PBMNCs have been implemented in several trials involving CLI patients, greatly expanding the achievements and possibilities of cell-based therapeutic angiogenesis [22–27]. In particular, these trials demonstrated the safety and feasibility of cell-based therapeutic angiogenesis for CLI patients (Table 1). However, it is still unlikely that this therapeutic strategy will fulfill the promise of a general use in clinical settings because of limited therapeutic outcomes.

**Table 1:** Clinical trials using BMMNCs and PBMNCs for CLI patients

Author	Year	Cell type	Target disease	Delivery	Study population	Outcome	Follow-up	References
Esato et al.	2002	BMMNC	CLI	IM	8	↑Symptom, ↑thermography, complete ulcer healing; 2/3 (67%) major amputation rates; 0	Not determined	[20]
Tateishi-Yuyama et al.	2002	BMMNC and PBMNC	CLI	IM	45	↑Symptom, ↑ABI, ↑TcPO2, complete ulcer healing; 6/10 (60%) major amputation rates; not shown	4 and 24 weeks	[25]
Huang et al.	2005	PBMNC	CLI	IM	28	↑Symptom, ↑ABI, ↑LDP, complete ulcer healing; 14/18 (78%) major amputation rates; 0	3 months	[88]
Lenk et al.	2005	PBMNC	CLI	IA	7	↑Symptom, ↑ABI, ↑TcPO2, complete ulcer healing; not shown major amputation rates; 0	20 weeks	[89]
Miyamoto et al.	2006	BMMNC	CLI	IM	8	↑Symptom, no ∆ ABI, complete ulcer healing; 7/8 (88%) major amputation rates; 0	684 days	[81]
Durdu et al.	2006	BMMNC	CLI	IM	28	↑Symptom, ↑ABI, ↑LDP, complete ulcer healing; 15/18 (83%) major amputation rates; 0	16.6 months	[79]
Arai et al.	2006	BMMNC	CLI	IM	39	↑Symptom, ↑ABI, ↑TcPO2, complete ulcer healing; 3/8 (38%) major amputation rates; not shown	1 months	[90]
Kawamoto et al.	2009	PBMNC (CD34+)	CLI	IM	17	↑Symptom, ↑TBI, ↑TcPO2, no ∆ ABI, complete ulcer healing; not shown major amputation rates; 0	3 months	[91]
Prochazka et al.	2010	BMC	CLI	IM	96	↑Symptom, ↑ABI, ↑LDP, ↑SPP, no ∆ TcPO2, complete ulcer healing; 33/42 (79%) major amputation rates; 9/42 (21%)	4 months	[92]
Murphy et al.	2011	BMMNC	CLI	IM	29	↑Symptom, ↑FTP, ↑TBI, complete ulcer healing; 3/9 (33%) major amputation rates; 4/29 (14%)	12 months	[93]
Walter et al.	2011	BMMNC	CLI	IA	40	↑Symptom, no ∆ ABI, no ∆ TcPO2, complete ulcer healing; 3/15 (20%) major amputation rates; 3/19 (16%)	3 months	[78]
Losordo et al.	2012	PBMNC (CD34+)	CLI	IM	28	No ∆ symptom, no ∆ ABI, no ∆ TBI, complete ulcer healing; 2/5 (40%) major amputation rates; 5/16 (31%)	12 months	[94]
Tanaka et al.	2014	PBMNC (CD34+)	CLI	IM	5	↑Symptom, ↑SPP, ↑TcPO2, no ∆ ABI, complete ulcer healing; 2/5 (40%) major amputation rates; 0	5 months	[95]
Teraa et al.	2015	BMMNC	CLI	IA	160	↑Symptom, ↑ABI, ↑TcPO2, complete ulcer healing; 19/51 (37%) major amputation rates; 21/81 (26%)	9 months	[96]

*BMMNC* bone marrow derived mononuclear cell, *PBMNC* peripheral blood mononuclear cell, *BMC* bone marrow cell, *CLI* critical limb ischemia, *IM* intramuscular, *IA* intraarterial, ↑ improved, ∆ change, *ABI* ankle brachial pressure index, *TcPO*_*2*_ transcutaneous oxygen pressure, *SPP* skin perfusion pressure, *LDP* laser Doppler perfusion, *TBI* toe brachial pressure index, *FTP* first toe pressure

In this review, we focus mainly on the challenges and limitations of cell-based therapeutic angiogenesis raised by previous studies, and discuss potential therapeutic strategies for its clinical application in CLI.

## Mechanism of cell-based therapeutic angiogenesis

In spite of yielding promising results, the mechanism of cell-based therapeutic angiogenesis remains vastly unknown. Cell-based therapeutic angiogenesis is thought to depend on a combination of secreted pro-angiogenic factors and direct differentiation of graft into vessel cells [28–30]. However, recent studies have suggested that a direct contribution of graft cells to the neovascularization of ischemic limbs is relatively rare. Instead, multiple pro-angiogenic factors secreted by graft cells are most likely responsible for the efficacy of therapeutic neovascularization [31–33].

VEGF, a dimeric glycoprotein of ~45 kDa, is an early pro-angiogenic factor in therapeutic angiogenesis [34]. VEGF binds to the FLT-1 and FLK-1 receptors on endothelial cells (ECs), activating their intracellular tyrosine kinases. This triggers phosphoinositide-3-kinase/Akt, and mitogen-activated protein kinase signaling pathways, promoting EC proliferation, migration, and survival [35, 36]. VEGF-A_165_, a VEGF isoform, binds also to the co-receptor neuropilin-1. In an initial clinical trial, in which the VEGF gene was delivered on a plasmid, the collateral formation of blood vessels was effectively induced in ischemic limbs [37].

Basic fibroblast growth factor (bFGF) is also a promising pro-angiogenic factor for therapeutic angiogenesis in CLI patients [9, 38]. The mechanism of action of bFGF in angiogenesis can be explained by the direct effect of FGF receptors on EC proliferation and migration [8]. Interestingly, bFGF contributes to angiogenesis in synergy with VEGF. A combination therapy with congenial pro-angiogenic factors represents a possible strategy for enhancing the effect of therapeutic angiogenesis in CLI patients [39].

Hepatocyte growth factor (HGF) also possesses angiogenic activity, which is exerted through phosphorylation of the tyrosine kinase of its specific receptor, c-Met, stimulating the motility and growth of ECs [40]. As with VEGF, direct delivery of HGF using plasmids has been tested on CLI patients in several clinical trials, demonstrating its safety and potential benefits during the early phase [41, 42].

Although the aforementioned pro-angiogenic factors act mainly on the motility of ECs to initiate vascular structures, it is thought that functional maturation of new vessels is required for the suitable recovery of blood flow in CLI patients. Platelet-derived growth factor-BB (PDGF-BB) recruits mural cells, also known as pericytes, and induces maturation of newly formed vessels [43]. Accordingly, a combination of cell-based therapeutic angiogenesis and PDGF-BB could represent an effective strategy for CLI patients.

## Source of graft cells for therapeutic angiogenesis

For example, mesenchymal stem cells (MSCs) and adipose-derived stem cells (ADSCs) are potential therapeutic sources of neovascularization because of their utilities in addition to angiogenic activity. Particularly, immune-privilege of MSCs has been paid attention for autologous transplantation [44]. However, it is still controversial which cell types are best for cell-based therapeutic angiogenesis in CLI patients. After investigating the therapeutic efficacy of various cell types in animal models and patients, mononuclear cells from bone marrow and peripheral blood (e.g., BMMNCs and PBMNCs) appear to be the most realistic choice in clinical settings. Common characteristics of these cell types are the presence of EPCs and the ability to secrete various pro-angiogenic factors. Although cellular heterogeneity and differentiation capacity vary between BMMNCs and PBMNCs, their clinical outcomes are not significantly different [21, 45, 46]. In fact, the major difference between these cells is represented by their invasiveness and isolation procedure. BMMNCs are collected from the iliac bone under general anesthesia, whereas PBMNCs are obtained from peripheral blood by leukapheresis without anesthesia. Minimal invasiveness and absence of anesthesia are required for high-risk CLI patients. Therefore, PBMNCs might be more suitable than BMMNCs for cell-based therapeutic angiogenesis in CLI patients, particularly given that the therapeutic effect is similar [21].

## Problems of cell-based therapeutic angiogenesis

Poor graft cell survival remains an unsolved problem for cell-based therapies in ischemic diseases. Reduced oxygen supply and high levels of inflammatory cytokines in ischemic tissues cause excessive production and consequent accumulation of reactive oxygen species, resulting in the death of graft cells [47, 48]. Declining cellular activities in elder patients may also contribute to reduced graft survival in ischemic tissues [49–52]. Therefore, to be effective, cell-based therapies should enhance tolerance against oxidative stress and the angiogenic potential of graft cells.

Another important problem in cell-based therapeutic angiogenesis is the maturation of newly formed vessels. These must be fully functional to supply sufficient blood flow to meet the oxygen and metabolic needs of ischemic tissues. However, newly formed vessels generated by cell transplantation are often immature, even if their number is generally sufficient [53]. Therefore, in addition to increasing the number of vessels, novel therapeutic strategies should also stimulate their maturation during neovascularization.

### Hypoxic pretreatment of graft cells to augment therapeutic potential

To enhance the efficacy of cell-based therapeutic angiogenesis, several approaches have been developed and tested in pre-clinical studies [54–56]. To this end, we and others have developed a “hypoxic preconditioning” method, whereby graft cells are incubated for a short time in low oxygen prior to cell transplantation.

**Fig. 1:**
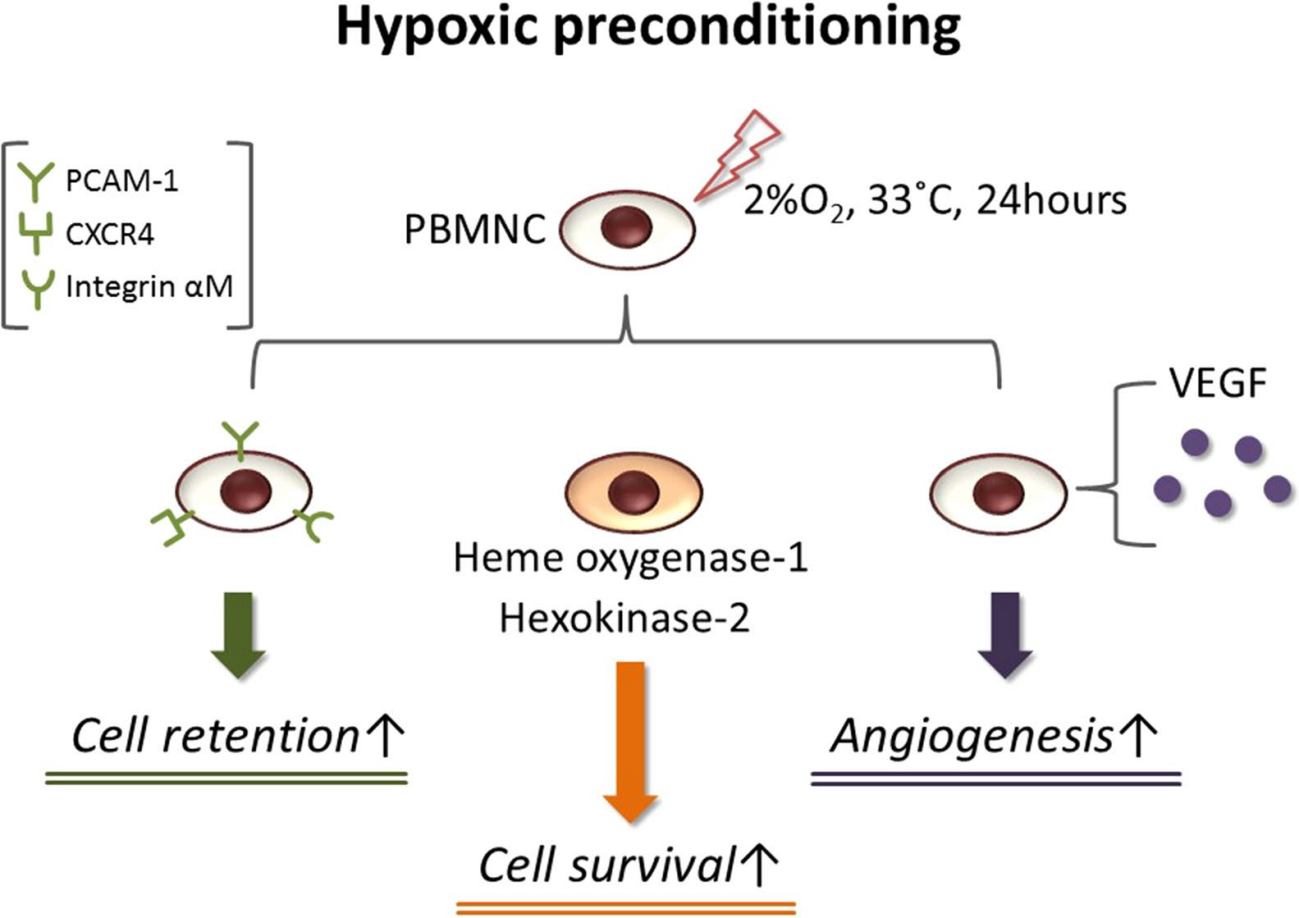
Schematic representation of hypoxic preconditioning of peripheral blood mononuclear cells (PBMNCs). Hypoxic preconditioning of PBMNCs at 2% O_2_ and 33 °C for 24 h. Cell retention, cell survival, and angiogenic potency are increased by this simple method, improving efficacy of cell-based therapy in ischemic conditions

Hypoxic preconditioning enhances VEGF production of mononuclear cells (MNCs) and EPCs, resulting in successful neovascularization in a rodent hind limb ischemia model [15, 57, 58]. In addition to angiogenic activity, hypoxic preconditioning affects also resistance to oxidative stress and adhesion of graft cells to ischemic tissues [59–62]. Such increases in cellular function in preconditioned cells result from upregulation of multiple gene sets associated with cell adhesion, stress resistance, and anti-apoptosis (Fig. 1). Interestingly, hypoxic preconditioning affects neovascularization even in MNCs of aged mice [63], suggesting that this method can, at least in part, reinforce “functionally-declined” MNCs. Moreover, hypoxic preconditioning augments the cellular functions of other cell types, including mesenchymal stem cells and engineered cell sheets [64, 65]. Taken together, given its simplicity and versatility, hypoxic preconditioning is one of most feasible “boosters” of cell-based therapy.

**Fig. 2:**
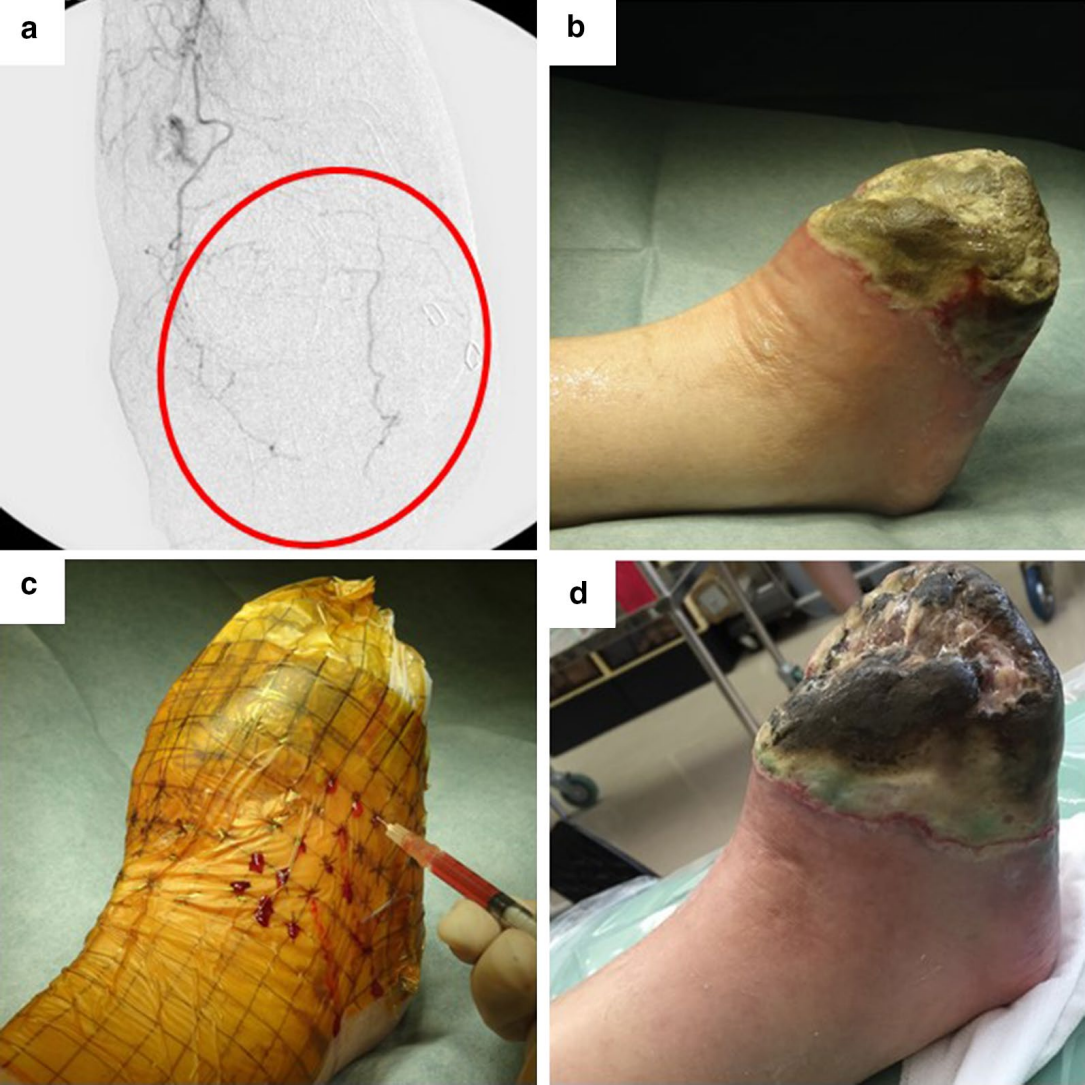
A patient with right foot atherosclerotic gangrene after injection of preconditioned cells. **a** The angiography revealed a poor vascular bed in the right foot (*circle*). **b** Location of the gangrene-infected amputation site of the Lisfranc in the right foot. Skin perfusion pressure (SPP) was 27 mm Hg pre-treatment. **c** Hypoxic preconditioned peripheral blood mononuclear cells were transplanted into 54 points (1 × 10^7^/0.1 mL/point) in ischemic tissue (5.4 × 10^8^ cells). **d** SPP increased to 59 mmHg 7 days after treatment, however necrosis and infection gradually worsened

Because hypoxic preconditioning is a simple but powerful method to enhance multiple cellular functions of MNCs, it can satisfy the need for therapeutic efficacy and rapidity strongly required in clinical settings. We have recently started a clinical trial using hypoxic preconditioning whereby autologous PBMNCs were transplanted into ischemic limbs of CLI patients. A CLI patient treated with preconditioned PBMNCs was thus relieved of severe ischemic pain and showed increased blood flow in the ischemic limb (*unpublished preliminary results*). Briefly, the patient was categorized as Rutherford class 6 and had undergone amputation of the Lisfranc because of remaining foot gangrene following several angioplasties. In spite of the initial amputation, strong ischemic pain and progressive necrosis remained in the foot. In the present trial, we aimed to release rest pain and stop the worsening of necrosis, in addition to checking the safety of this therapeutic approach. As a result, the patient, who was injected with 5.4 × 10^8^ preconditioned PBMNCs into the ischemic leg, was released from rest pain. Skin perfusion pressure increased (from 27 to 59 mmHg) and there were no adverse events. However, the CLI patient, who injected cells, had to be re-amputated above the ankle a month after cell transplantation because of uncontrollable necrosis and infection of the gangrenous foot (Fig. 2).

Consistent with this study, some trials have reported that the therapeutic effects of cell-based therapies were not as expected in CLI patients with diabetes mellitus, hemodialysis, and advanced Rutherford class 6 [66–68]. Therefore, it is important to determine the correct indication and adequate timing of cell-based therapies. In addition, we believe that a more powerful therapeutic strategy is necessary for high-risk patients.

### Combination therapy to induce new vessels and their maturation

Evidence from preclinical studies using multi-growth factors supports the notion that a combination of induction and maturation of new vessels improves functional outcomes of therapeutic angiogenesis even in CLI patients [39, 69–72]. As mentioned previously, cell-based therapy is a promising strategy to induce new vessels in ischemic tissues, including CLI, and hypoxic preconditioning is a possible booster to enhance therapeutic angiogenesis. Therefore, a combination of cell transplantation that includes hypoxic preconditioning, and the use of vessel maturation-associated factors might provide a novel effective therapeutic strategy for CLI.

**Fig. 3:**
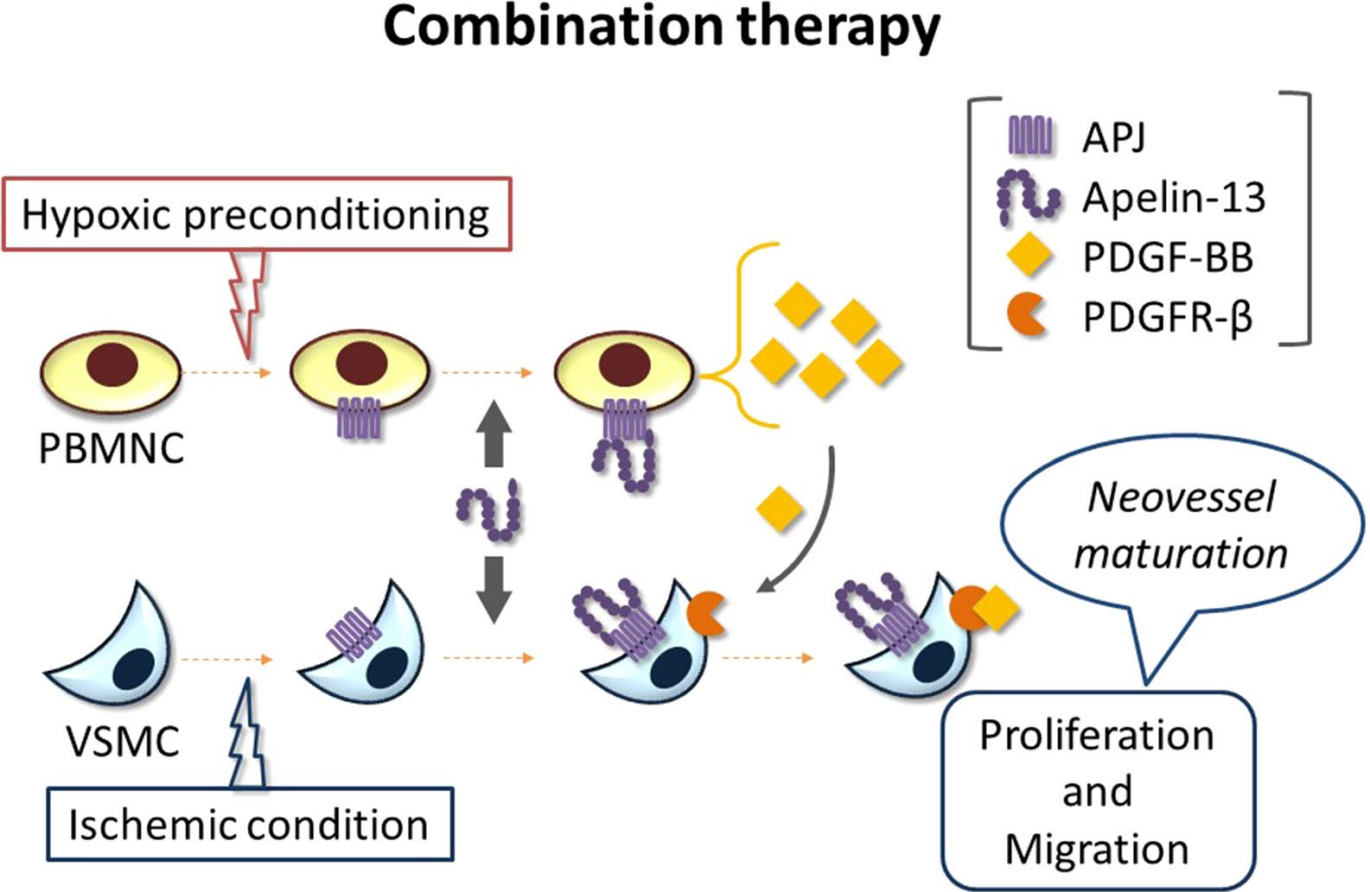
Combination therapy using hypoxic preconditioning and apelin. Hypoxic preconditioning and ischemic conditions upregulate the APJ receptor in peripheral blood mononuclear cells (PBMNCs) and vascular smooth muscle cells (VSMCs). Preconditioned PBMNCs receive exogenous apelin, leading to the secretion of platelet-derived growth factor (PDGF)-BB. Exogenous apelin induces upregulation of PDGF receptor-β in ischemic VSMCs. Subsequently, VSMCs are activated to mature newly formed vessels in ischemic tissue

Angiopoietin-1 (Ang-1) and apelin are well known as vessel maturation-associated factors. Apelin, which is an endogenous ligand for the APJ receptor, regulates caliber size and stabilization of blood vessels; whereas Ang-1 contributes to EC migration during vessel maturation [73–76]. Recently, we investigated whether a combination of preconditioned cell transplantation and apelin administration could represent an effective therapeutic strategy for CLI. We found that hypoxic preconditioning enhanced the sensitivity of PBMNCs to apelin through upregulation of the APJ receptor, thereby resulting in increased PDGF-BB secretion. At the same time, apelin directly regulated proliferation and migration of vascular smooth muscle cells in ischemic blood vessels through induction of PDGF receptor-β (Fig. 3). Thus, a combination of preconditioned cell transplantation and apelin administration induced functionally matured new vessels and dramatically improved blood flow to the ischemic hind limbs in CLI animal models [53]. Our findings raise the possibility that cell-based therapeutic angiogenesis may benefit from the combined administration of vessel maturation-associated factors.

## Possible targets of cell-based therapeutic angiogenesis in CLI

Some clinical trials show discrepancies in the therapeutic outcomes of cell-based therapeutic angiogenesis among CLI patients. For example, the therapeutic angiogenesis using cell transplantation (TACT) trial, which was performed in patients with TAO using BMMNCs, demonstrated long-term safety and a higher therapeutic efficacy than in ASO patients [77]. Similarly, the PROVASA (intraarterial progenitor cell transplantation of bone marrow mononuclear cells for induction of neovascularization in patients with peripheral arterial occlusive disease) trial indicated greater overall therapeutic benefits in TAO compared with atherosclerotic CLI patients [78]. Moreover, other trials have also demonstrated a more efficient outcome of cell-based therapeutic angiogenesis in TAO than in ASO patients [79–82]. Such clinical evidence suggests that some targets may be more appropriate than others, although it remains to be determined why cell-based therapeutic angiogenesis is more effective in TAO than in ASO patients. Given that TAO is defined as a non-atherosclerotic and inflammatory disease, whereas ASO is associated with atherosclerosis and advanced age [49, 50, 83], these pathologies might determine the outcomes of cell-based therapeutic angiogenesis in CLI patients. Accordingly, we may be able to find targets other than TAO for effective cell-based therapeutic angiogenesis in CLI patients.

Similar pathological characteristics, ranging from inflammation of small- and middle-size arteries to TAO, are observed also in patients with collagen vascular diseases (CVDs). It is thought that auto-immune disorders are underlying diseases commonly associated with both TAO and CVD. Patients with CVD present symptoms of vasculitis and occlusion of microvessels, resulting in rest pain, skin ulcer, and gangrene in the limbs. In spite of many attempts to find a cure, there are no effective drugs against CVD. Given the absence of a vascular bed in microcirculatory systems of the extremities, surgical treatments including bypass surgery do not provide adequate blood flow to ischemic limbs for long periods of time [84]. If cell transplantation could provide a vascular bed in ischemic limbs, then cell-based therapeutic angiogenesis would be a reasonable therapeutic strategy for untreatable CVD patients. A possible application of cell-based therapeutic angiogenesis for patients with CVD has been reported in some clinical trials [85–87]. Taken together, cell-based therapeutic angiogenesis could become a powerful tool in CLI with inflammation and a poor vascular bed. However, further investigation is required to ensure this therapeutic approach is translated into the right practical applications.

## Conclusion

Efficacy and safety of cell-based therapeutic angiogenesis have been demonstrated in many clinical trials. However, therapeutic outcomes are still limited and further improvements are required for extensive clinical applications. For example, hypoxic preconditioning of graft cells and its combination with other strategies are some of the options for enhancing efficacy of cell-based therapeutic angiogenesis. Also, absence of necrosis and infection at the time of cell injection, and an appropriate selection of target diseases, such as TAO and vascular diseases caused by auto-immune disorders, should be considered when translating this approach to clinical settings.

## Abbreviations

ADSCs: adipose-derived stem cells
ASO: atherosclerosis obliterans
bFGF: basic fibroblast growth factor
BMMNCs: bone marrow-derived mononuclear cells
CLI: critical limb ischemia
CVD: collagen vascular disease
ECs: endothelial cells
EPCs: endothelial progenitor cells
HGF: hepatocyte growth factor
MNCs: mononuclear cells
MSCs: mesenchymal stem cells
PAD: peripheral artery disease
PBMNCs: peripheral blood mononuclear cells
PDGF: platelet-derived growth factor
TAO: thromboangiitis obliterans
VEGF: vascular endothelial growth factor
